# Targeted Therapies in Generalized Myasthenia Gravis: An Umbrella Review of Evidence Quality and Overlap

**DOI:** 10.3390/neurosci7050107

**Published:** 2026-09-30

**Authors:** Felipe Castillo, Macarena Morel-Marambio, Francisco Pérez-Mora, Luis Ortíz-Muñoz, Gabriel Bustamante, Francisca Fajre, Gabriel Cea

**Affiliations:** 1 Master’s Program Student in Health Sciences Research, Escuela de Medicina, Pontificia Universidad Católica de Chile, Santiago 8330077, Chile; 2 Departamento Médico, AstraZeneca, Santiago 7550106, Chile; 3 Evidencia UC, Pontificia Universidad Católica de Chile, Santiago 8331150, Chile; juana.morell@uc.cl (M.M.-M.); leortizmunoz@uc.cl (L.O.-M.); 4 Centro de Investigación en Salud Pública y Epidemiología Clínica (CISPEC), Facultad de Ciencias de la Salud Eugenio Espejo, Universidad UTE, Quito 170527, Ecuador; 5 Departamento de Neurología, Pontificia Universidad Católica de Chile, Santiago 7820436, Chile; gubustamante@uc.cl; 6 Departamento de Neurología, Hospital Dirección de Previsión de Carabineros de Chile (DIPRECA), Santiago 7601003, Chile; 7 Departamento de Neurología, Hospital San Juan de Dios, Santiago 8350488, Chile; 8 Departamento de Ciencias Neurológicas Oriente, Facultad de Medicina, Universidad de Chile, Santiago 7500922, Chile; jcea@uchile.cl; 9 Servicio de Neurología, Hospital del Salvador, Servicio de Salud Metropolitano Oriente, Santiago 7500922, Chile

**Keywords:** myasthenia gravis, complement inhibitors, FcRn antagonists, rituximab, umbrella review

## Abstract

Generalized myasthenia gravis (gMG) is an antibody-mediated autoimmune disorder in which complement C5 inhibitors and neonatal Fc receptor (FcRn) antagonists have expanded therapeutic options, while rituximab remains used off-label in selected patients. We conducted an umbrella review to critically appraise systematic reviews and evidence syntheses evaluating complement inhibitors and/or FcRn antagonists within a therapeutic framework that also included rituximab. MEDLINE/PubMed, Embase, Epistemonikos, Google Scholar, and supplementary citation sources were searched from 2015 onward, with an updated search on 15 August 2026. Methodological quality was assessed with AMSTAR 2 and primary-study overlap with the corrected covered area (CCA) and GROOVE. Ten reviews/evidence syntheses met eligibility criteria. Complement inhibitors and FcRn antagonists were generally associated with improvements in MG-ADL and QMG, although estimates and rankings varied. AMSTAR 2 rated one review as high confidence, four as low confidence, and five as critically low confidence. Primary-study overlap was very high (CCA 32.59%), with 36 of 45 pairwise comparisons classified as very high. Comparative evidence, including rituximab, was predominantly indirect. Targeted therapies provide clinically relevant benefit. The protocol was registered with the Open Science Framework (OSF, 27 June 2025).

## 1. Introduction

Myasthenia gravis (MG) is a rare autoimmune disease in which antibodies bind to acetylcholine receptors or to functionally related molecules in the postsynaptic membrane at the neuromuscular junction [1]. When muscle weakness is not confined to the extraocular muscles, the condition is termed generalized myasthenia gravis (gMG), characterized by fluctuating skeletal muscle weakness that worsens with activity and improves with rest.

MG can be classified according to the presence of serum autoantibodies. Antibodies against the acetylcholine receptor (AChR) are detected in approximately 80–85% of patients with generalized MG, whereas muscle-specific kinase (MuSK) antibodies are found in around 5–8%. Antibodies against low-density lipoprotein receptor-related protein 4 (LRP4) have also been described in a smaller and more variable proportion of patients, particularly among those negative for AChR and MuSK antibodies. A subset of patients remains negative for AChR and MuSK antibodies using conventional assays and is commonly referred to as double-seronegative MG; however, the proportion of seronegative patients varies depending on the antibodies tested and the diagnostic methods used [2,3,4,5].

The standard therapeutic approach for MG has relied on acetylcholinesterase inhibitors, corticosteroids, non-steroidal immunosuppressants such as azathioprine and mycophenolate mofetil, and thymectomy in selected patients, particularly those with thymoma or non-thymomatous AChR-positive generalized MG [6]. The benefit of thymectomy in non-thymomatous AChR-positive generalized MG is supported by randomized evidence showing improved clinical outcomes and reduced corticosteroid requirements when thymectomy was added to prednisone [7]. Although these therapies are effective in improving MG-related symptoms in many patients, they may carry a considerable burden of long-term adverse effects [6,8]. Moreover, up to 15% of individuals with MG exhibit limited or no response to these standard therapies [9] and may be classified as refractory (or highly active) disease, typically defined by persistent clinically relevant symptoms with MGFA post-intervention status unchanged or worse despite adequate trials of corticosteroids and ≥2 other immunosuppressive agents, or by treatment-limiting intolerance [9,10]. These patients often experience recurrent exacerbations, crisis-like deterioration, and a substantial treatment burden [10].

In recent years, rituximab (RTX), a chimeric anti-CD20 monoclonal antibody that depletes circulating B cells, has been used off-label in selected patients with refractory or highly active MG. Its therapeutic effect appears to differ according to antibody-defined disease subtype. Evidence has been particularly favorable in MuSK-antibody-positive MG, whereas outcomes in AChR-antibody-positive gMG have been more heterogeneous. In AChR-Ab+ disease, randomized evidence has not consistently demonstrated a clear steroid-sparing benefit, although clinically meaningful responses have been reported in selected patients [11,12]. These therapeutic limitations, together with advances in the understanding of MG pathophysiology, have driven the development of mechanism-based therapies targeting B cells, the complement cascade, the neonatal Fc receptor (FcRn), and other immune pathways [13].

IgG1 antibodies associated with AChR-Ab+ MG can activate the classical complement pathway, leading to formation of the membrane attack complex and structural damage at the postsynaptic membrane of the neuromuscular junction [14]. Eculizumab is a recombinant humanized monoclonal antibody that binds complement component C5, preventing its cleavage into C5a and C5b and thereby inhibiting terminal complement activation and membrane attack complex formation. In the pivotal phase 3 REGAIN trial in patients with refractory AChR-Ab+ generalized MG, eculizumab did not meet the prespecified primary endpoint, as the change in MG-ADL score was not statistically significant versus placebo using the worst-rank analysis; however, clinically relevant improvements were observed across several secondary outcomes and subsequent analyses [15]. Ravulizumab, a long-acting C5 inhibitor engineered from eculizumab, was subsequently evaluated in the phase 3 CHAMPION MG trial in adults with AChR-Ab+ generalized MG, showing significantly greater improvements versus placebo from baseline to week 26 in MG-ADL and QMG total scores [16]. Zilucoplan, a 15-amino-acid macrocyclic peptide that binds C5 with high affinity and inhibits both C5 cleavage and terminal complement activation, was evaluated in the phase 3 RAISE trial and showed a significantly greater reduction in MG-ADL score from baseline to week 12 compared with placebo in patients with AChR-Ab+ generalized MG [17]. Ravulizumab and zilucoplan met their primary MG-ADL endpoints in phase 3 trials, with placebo-adjusted LS mean differences of −1.6 points at week 26 in CHAMPION MG and −2.09 points at week 12 in RAISE, respectively. These C5-targeted therapies have obtained regulatory approval for AChR-Ab+ generalized MG, although approved indications differ across regions and products.

The neonatal Fc receptor (FcRn) plays a crucial role in protecting circulating IgG from lysosomal degradation; therefore, FcRn blockade accelerates IgG catabolism and reduces pathogenic IgG autoantibody levels. Efgartigimod, an engineered human IgG1 Fc fragment administered intravenously, binds FcRn with increased affinity and prevents IgG recycling. In the phase 3 ADAPT trial, efgartigimod was associated with clinically meaningful improvement in AChR-Ab+ generalized MG, with a higher proportion of MG-ADL responders than placebo during the first treatment cycle (68% vs. 30%) [18]. Rozanolixizumab, a humanized IgG4 monoclonal antibody targeting FcRn and administered subcutaneously, also showed significant improvements in MG-specific outcomes in the phase 3 MycarinG trial, with placebo-adjusted MG-ADL least-squares mean differences of −2.59 for the 7 mg/kg dose and −2.62 for the 10 mg/kg dose at day 43 [19]. Batoclimab, a humanized IgG1 monoclonal antibody against FcRn administered subcutaneously, showed significant improvement in MG-ADL in a phase 3 trial, with an approximate placebo-adjusted difference of −1.9 points; however, it is not currently FDA-approved for generalized MG [20]. More recently, nipocalimab, a human FcRn-blocking monoclonal antibody administered intravenously, demonstrated sustained improvement in MG-ADL in the phase 3 Vivacity-MG3 trial, with an approximate placebo-adjusted difference of −1.45 points over weeks 22–24, and has received FDA approval for anti-AChR- or anti-MuSK-antibody-positive generalized MG in patients aged 12 years and older [21].

The rapid expansion of targeted therapies in gMG has been accompanied by a growing number of systematic reviews and network meta-analyses evaluating their efficacy, safety, and relative therapeutic effects. However, these evidence syntheses have yielded variable conclusions regarding treatment rankings and comparative efficacy, while often relying on the same underlying randomized trials. Differences in antibody-defined populations, disease severity, previous treatments, outcome definitions, and follow-up periods may further limit the comparability of treatment effects across reviews. Assessing methodological quality and primary-study overlap is therefore essential to determine how independent and reliable this rapidly expanding secondary evidence actually is.

Within this evidence landscape, rituximab was considered a relevant therapeutic context because of its established off-label use in gMG and its inclusion in comparative evidence syntheses. Accordingly, this umbrella review intentionally focused on systematic reviews and meta-analyses in which complement inhibitors and/or FcRn antagonists were evaluated within a therapeutic framework that also included rituximab. Direct head-to-head comparison with rituximab was not required. The aim was to critically appraise the available secondary evidence, with particular emphasis on methodological quality, primary-study overlap, and the limitations of comparative treatment estimates in adults with gMG.

## 2. Materials and Methods

We conducted an umbrella review in accordance with Joanna Briggs Institute (JBI) guidance, North Adelaide, Australia [22]. This umbrella review was reported according to the Preferred Reporting Items for Systematic Reviews and Meta-Analyses (PRISMA) guidelines [23]. The protocol was registered in the Open Science Framework (OSF) https://osf.io/e89vp (accessed on 21 April 2025).

### 2.1. Search Strategy

Databases searched included MEDLINE (via PubMed), Embase, and Epistemonikos. Google Scholar was used as an additional source for citation searching and identification of potentially relevant grey literature. Systematic reviews and meta-analyses published from 1 January 2015 onward were eligible. The original database searches were conducted between 21 and 25 April 2025. As more than 12 months had elapsed since the original search, an updated search was conducted on August 15, 2026, using the same databases, predefined search strategy, and eligibility criteria to ensure that the evidence base remained current [24]. No language restrictions were applied.

Controlled vocabulary and free-text terms were adapted to the indexing and syntax of each database (e.g., MeSH terms in PubMed and Emtree terms in Embase). The main concepts were (“myasthenia gravis” OR “generalized myasthenia gravis”) AND (“complement inhibitors” OR eculizumab OR ravulizumab OR zilucoplan OR “FcRn antagonists” OR “FcRn blockers” OR efgartigimod OR rozanolixizumab OR nipocalimab OR batoclimab) AND rituximab. Additional commercial names included Soliris, Ultomiris, Zilbrysq, Vyvgart, and Rystiggo. Boolean operators were used to combine synonyms and concepts.

Rituximab was intentionally included as a mandatory search concept because the purpose of this umbrella review was not to identify all systematic reviews of targeted therapies in gMG, but specifically those in which complement inhibitors and/or FcRn antagonists were evaluated within a therapeutic evidence framework that also included rituximab. Direct head-to-head comparisons with rituximab were not required for eligibility.

The complete search strategy can be found in Supplementary Text S1. Filters for systematic reviews and meta-analyses were applied where available. Each search was documented, and all retrieved records were exported to a Zotero library as RIS files.

### 2.2. Eligibility Criteria and Types of Studies

#### 2.2.1. Inclusion Criteria

The PICO (Population, Intervention, Comparator, and Outcome) criteria below defined the scope of our umbrella review:

Population: Adults aged 18 years and older diagnosed with generalized MG (restricted to adults because most pivotal trials and resulting evidence syntheses enrolled adult populations; juvenile MG is relatively uncommon).

Interventions: Complement inhibitors (eculizumab, ravulizumab, or zilucoplan) or FcRn blockers/antagonists (efgartigimod, rozanolixizumab, nipocalimab, batoclimab).

Comparator/contextual treatment: Rituximab, when included within the therapeutic evidence framework of the systematic review or meta-analysis. Direct head-to-head comparisons with rituximab were not required for eligibility. Rituximab was considered an indirect comparator when it contributed to comparative analyses through a common comparator or treatment network, and a contextual therapeutic option when it was included within the therapeutic framework without contributing a direct comparative estimate against the prespecified targeted therapies.

Outcomes: Myasthenia Gravis Activities of Daily Living (MG-ADL), Quantitative Myasthenia Gravis (QMG), Myasthenia Gravis Composite (MGC), 15-item revised Myasthenia Gravis Quality of Life (MG-QOL15r), or MGFA Post-Intervention Status (MGFA-PIS).

#### 2.2.2. Exclusion Criteria

We excluded any study design or report that was not a systematic review or meta-analysis (e.g., narrative reviews, observational studies, case reports, letters).

We excluded studies including patients with juvenile myasthenia gravis, ocular myasthenia gravis, neonatal myasthenia gravis, congenital myasthenic syndrome, or Lambert–Eaton myasthenic syndrome.

### 2.3. Study Selection

The search for published studies was conducted independently by two reviewers (FC and FP-M). After duplicates were removed, the search results were screened against the inclusion and exclusion criteria by the same reviewers (Supplementary Text S2). Titles and abstracts were screened first, followed by full-text screening. Rayyan [25] was used as the screening tool, facilitating blinded screening by multiple independent reviewers [26]. Each reviewer screened all records independently, and disagreements were resolved by a third reviewer (FF).

After completing the database searches, we conducted a second round of secondary searches. A citation search (“snowballing”) was performed in Google Scholar, and the first five pages of results were reviewed. In addition, Research Rabbit (https://www.researchrabbit.ai/ (accessed on 25 April 2025) was used to identify related articles and authors [27]. These two searches were conducted independently by two reviewers (FC and FP-M) (Supplementary Text S3).

The final list of included systematic reviews was checked by three myasthenia gravis experts (GB, FF, and GC). We also documented the records excluded during the original search, including reasons for exclusion (Supplementary Table S1).

### 2.4. Data Collection/Extraction

Data extracted included author and year, objective(s), participant characteristics and total sample size, MG antibody status (AChR, MuSK, LRP4, seronegative, mixed, or not reported), setting/context, date range of included studies, number of included studies, study designs, countries of origin, appraisal tool and rating, review type and analytical methods, outcomes, main results/findings, and reviewer comments (Supplementary Table S2). For each included review, the presence and role of rituximab were assessed based on the reported treatment network, comparative analyses, and therapeutic framework, and were categorized as an indirect comparator or contextual therapeutic option.

For each included review, outcome data were extracted as reported by the systematic review for the prespecified outcomes of interest. Primary-study reports were consulted only when necessary to clarify or verify incompletely reported study characteristics; no de novo treatment-effect estimates were derived from primary studies. Two reviewers independently performed data extraction.

### 2.5. Assessment of Methodological Quality

The methodological quality of reviews meeting the inclusion criteria was assessed using AMSTAR 2 (A Measurement Tool to Assess Systematic Reviews) [28]. AMSTAR 2 comprises 16 domains and is a popular instrument for critically evaluating the quality of systematic reviews of randomised and non-randomised controlled studies of healthcare interventions.

Two authors (F.C. and F.P.-M.) independently applied the checklist to all eligible reviews. Disagreements were resolved by consensus, with input from myasthenia gravis experts (G.B., F.F., and G.C.) when needed (Supplementary Table S3).

Based on the number of weaknesses in critical and non-critical domains, the overall confidence in each review was rated as high (no or one non-critical weakness), moderate (more than one non-critical weakness), low (one critical flaw with or without non-critical weaknesses), or critically low (more than one critical flaw with or without non-critical weaknesses).

### 2.6. Assessment of Study Overlap

We applied the corrected covered area (CCA) approach using the Graphical Representation of Overlap for Overviews (GROOVE) to assess whether overlapping primary studies might have influenced the findings of our umbrella review [29]. Supplementary Table S4.

We constructed an evidence matrix to evaluate overlap of primary studies across included systematic reviews (SRs). In this matrix, columns represented the included SRs and rows represented the primary studies within each SR. We considered only primary studies that contributed relevant data to our review questions. We then calculated the overall CCA and interpreted overlap as follows: <5% (slight), 5% to <10% (moderate), 10% to <15% (high), and ≥15% (very high) [30].

## 3. Results

**Figure 1:**
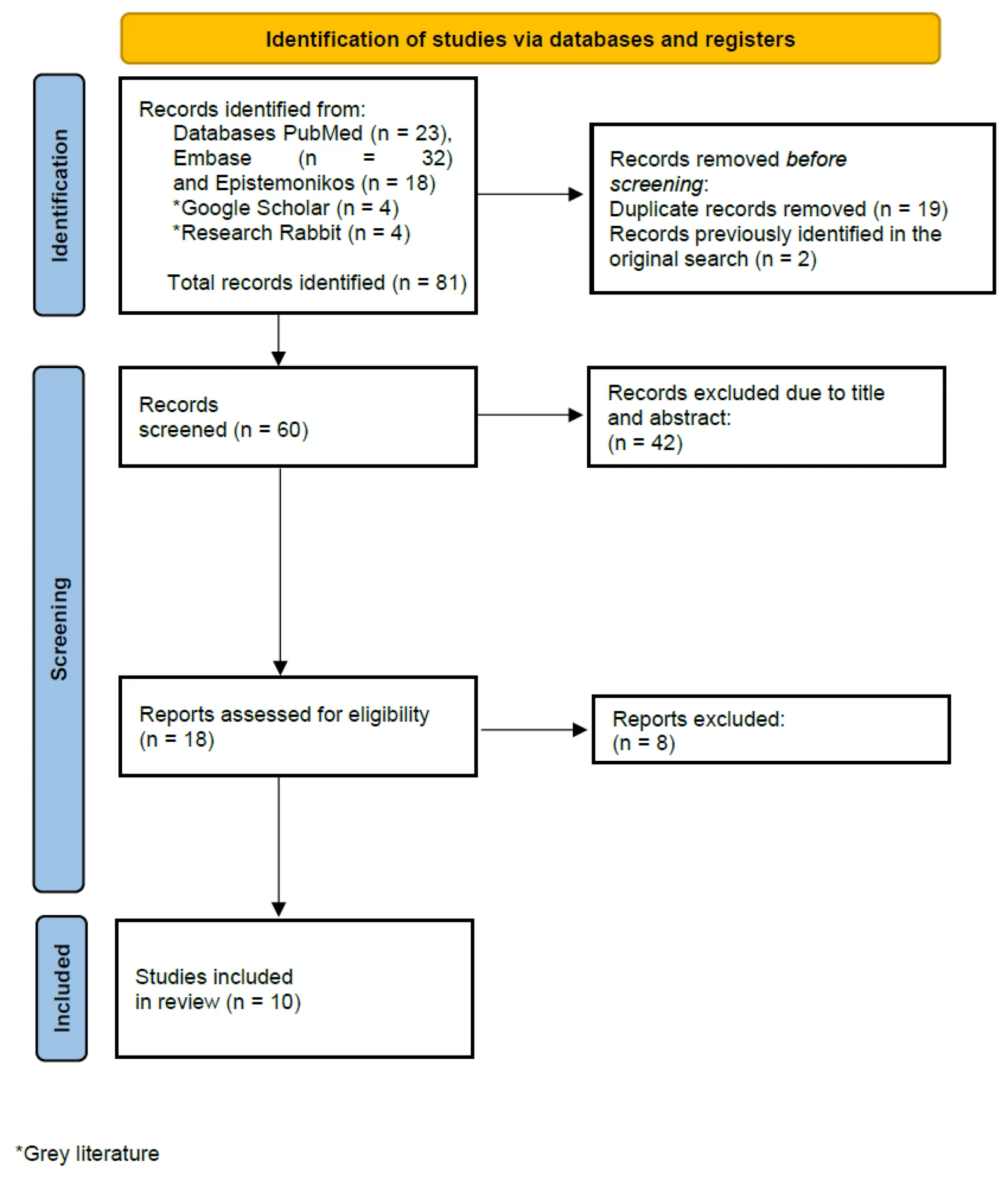
PRISMA flow diagram of the study selection process.

The original search identified 58 records: 51 from databases (MEDLINE/PubMed *n* = 15, Embase *n* = 21, Epistemonikos *n* = 15) and 7 from manual searches (Google Scholar *n* = 3 [31,32,33] and Research Rabbit *n* = 4 [34,35,36,37]). The updated search conducted on 15 August 2026 retrieved 23 additional records, including 22 from the electronic databases and one from Google Scholar. Across the original and updated searches, 81 records were identified. After removal of 19 duplicates and 2 records that had already been identified in the original search, 60 records underwent title and abstract screening. Forty-two records were excluded at this stage, and 18 reports were assessed for eligibility by full-text review. Eight reports were excluded after full-text assessment, resulting in 10 systematic reviews/evidence syntheses being included in the umbrella review (Figure 1).

The 10 included reviews evaluated complement inhibitors and/or FcRn-targeted therapies within therapeutic evidence frameworks that also included rituximab [31,38,39,40,41,42,43,44,45,46]. Across the 10 included reviews, rituximab was included as an indirect comparator in nine reviews and as a contextual therapeutic option in one ITC feasibility assessment. No direct head-to-head comparison between rituximab and the prespecified complement inhibitors or FcRn antagonists was identified. Eight reviews incorporated network meta-analysis, one was a systematic review and meta-analysis, and one systematic literature review was embedded within an indirect treatment comparison (ITC) feasibility assessment. Several reviews evaluated broader therapeutic networks that included additional immunotherapies; however, only evidence relevant to the prespecified interventions and outcomes of this umbrella review was extracted. Across the included reviews, the most frequently evaluated efficacy outcomes were MG-ADL and QMG, followed by MGC and MG-QOL15r. Antibody-defined populations were predominantly AChR-antibody positive, although some reviews also included MuSK-antibody-positive and seronegative patients.

Across the included quantitative evidence syntheses, complement inhibitors and FcRn-targeted therapies were generally associated with improvements in functional outcomes compared with placebo or standard care, although the magnitude and certainty of benefit varied across agents, outcomes, and reviews. For eculizumab, evidence syntheses reflected the findings of the pivotal REGAIN trial, which did not meet its prespecified primary MG-ADL endpoint using the worst-rank analysis, although several secondary, sensitivity, and longer-term analyses favored treatment. In contrast, ravulizumab demonstrated significant improvement in MG-ADL versus placebo in CHAMPION MG, with improvement emerging early and maintained through week 26. Among FcRn-targeted therapies, efgartigimod demonstrated clinically meaningful benefit in ADAPT, including a higher proportion of MG-ADL responders compared with placebo, while subsequent evidence supported sustained benefit with repeated treatment cycles. Similar improvements in MG-ADL and QMG were reported across evidence syntheses evaluating rozanolixizumab, nipocalimab, batoclimab, and zilucoplan. Comparative evidence involving rituximab remained predominantly indirect.

The three additional evidence syntheses identified in the updated search expanded, but also further highlighted the limitations of, the comparative evidence base. Waseem et al. included 18 randomized controlled trials in a network meta-analysis [44], whereas McLaren et al. identified 32 eligible prospective multi-arm studies, with 27 placebo-controlled randomized trials contributing to the principal network meta-analysis [45]. Gilhus et al. differed methodologically from the quantitative syntheses: their systematic literature review was used to assess the feasibility of indirect treatment comparisons by examining cross-trial heterogeneity, network connectivity, and differences in study design, populations, and outcome assessment rather than generating a new pooled comparative efficacy estimate [46].

### 3.1. Methodological Quality (AMSTAR 2)

Using AMSTAR 2, one systematic review was rated as high confidence (Chen, 2023) [41], four as low confidence (Gu, 2024; Feng, 2021; Guan, 2025; Gilhus, 2025) [31,40,42,46], and five as critically low confidence (Ma, 2024; Zhong, 2024; Saccà, 2023; Waseem, 2026; McLaren, 2026) [38,39,43,44,45] (Supplementary Table S3). Across the AMSTAR 2 critical domains, the most frequent limitation was the absence of a list of excluded studies with reasons (Domain 7: 8/10 reviews rated “No”). Protocol registration and prespecification (Domain 2) were rated “Yes” in 5/10 reviews, “Partial Yes” in 2/10, and “No” in 3/10. Search strategy comprehensiveness (Domain 4) was rated “Yes” in 4/10 reviews and “Partial Yes” in 6/10. Risk-of-bias assessment of primary studies (Domain 9) was rated “Yes” in 8/10 reviews and “Partial Yes” in 2/10. Appropriate meta-analytic methods (Domain 11) were rated “Yes” in 9/10 reviews and were not applicable in one review that did not perform a meta-analysis. Consideration of risk of bias when interpreting the findings (Domain 13) was rated “Yes” in 7/10 reviews and “No” in 3/10. Publication-bias assessment (Domain 15) was rated “Yes” in 6/10 reviews, “No” in 3/10, and was not applicable in one review without quantitative synthesis. Reporting of funding sources for the primary studies was limited (Domain 10: 9/10 reviews rated “No”).

### 3.2. Overlap (GROOVE/CCA)

**Figure 2:**
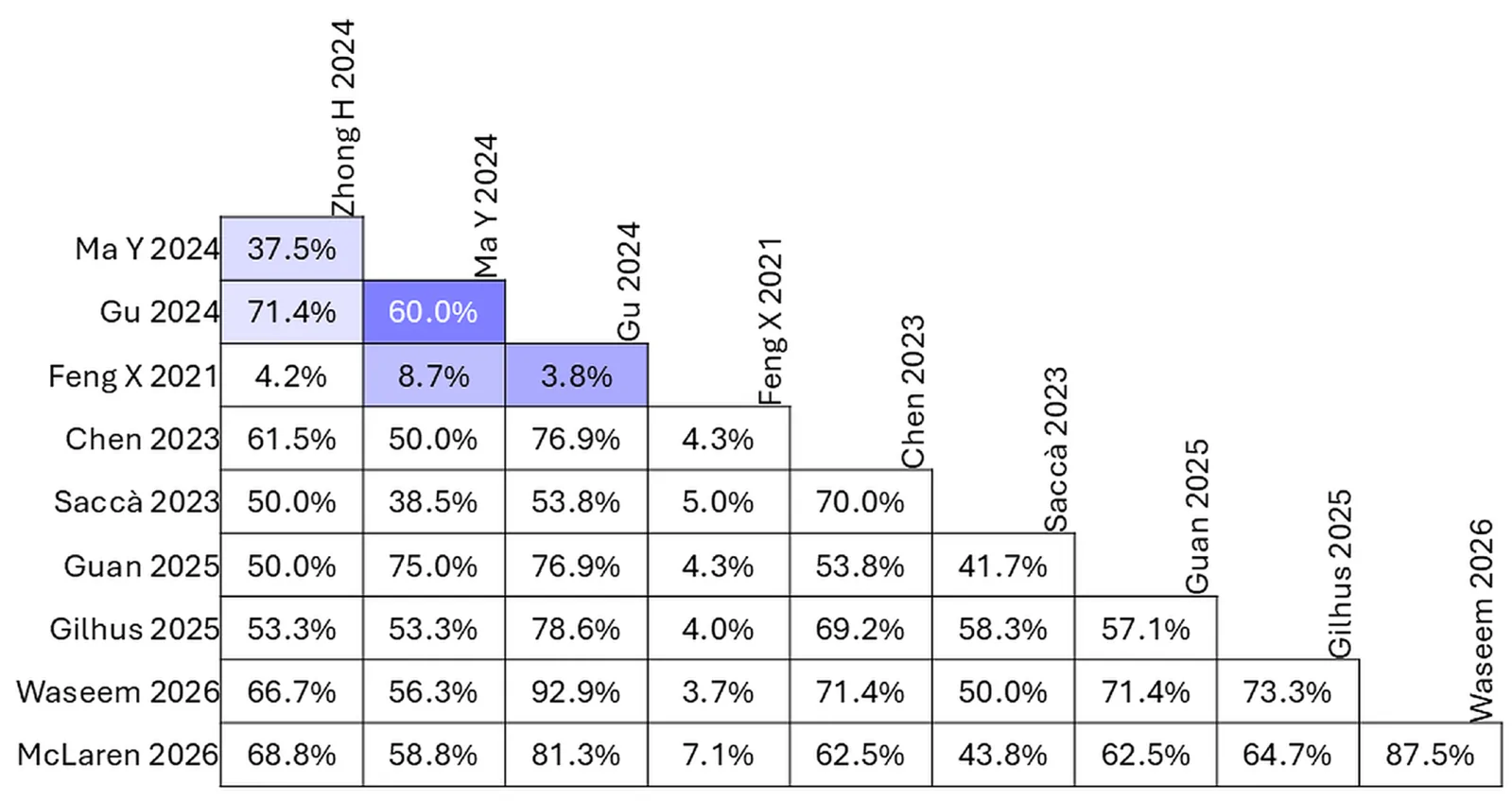
Overlap matrix of primary studies across systematic reviews included in the overview. Pairwise overlap of primary studies across systematic reviews included in the umbrella review. Cells show the percentage of shared primary studies between each pair of reviews. Systematic reviews represented in the matrix: Zhong et al. [43], Ma et al. [39], Gu et al. [40], Feng et al. [42], Chen et al. [41], Saccà et al. [38], Guan et al. [31], Gilhus et al. [46], Waseem et al. [44], and McLaren et al. [45]. Cells show the percentage of shared primary studies between each pair of reviews.

Overlap of primary studies across the included systematic reviews was quantified using the Graphical Representation of Overlap for Overviews (GROOVE) and the corrected covered area (CCA) method (Supplementary Table S4). Ten reviews (c = 10) were mapped against 30 unique index publications (r = 30), with 118 primary-study occurrences including double counting (N = 118), resulting in a covered area of 39.33%. The overall CCA was 32.59%, indicating a very high degree of overlap. No structural zeros were identified (X = 0); therefore, the CCA adjusted for structural zeros was unchanged at 32.59% (Table 1). Across the 45 pairwise review comparisons, overlap was classified as slight (<5%) in 6 pairs, moderate (5% to <10%) in 3 pairs, high (10% to <15%) in none, and very high (≥15%) in 36 pairs (Figure 2). These findings indicate substantial reuse of the same primary evidence across the included reviews.

## 4. Discussion

The results of this umbrella review suggest that targeted therapies are associated with clinically relevant improvements in functional outcomes in adults with gMG. Across the included systematic reviews and evidence syntheses (Supplementary Table S2), complement C5 inhibition and FcRn blockade were generally associated with improvements in patient-relevant outcomes, particularly MG-ADL, QMG, and MGC. However, the certainty and comparability of these findings were constrained by heterogeneous methodological quality, substantial overlap of primary studies across reviews, and the predominantly indirect nature of the comparative evidence.

Some network meta-analyses suggested differences in probabilistic treatment rankings across outcomes, with FcRn antagonists such as batoclimab and rozanolixizumab ranking favorably for MG-ADL, QMG, or MGC in certain analyses, whereas eculizumab ranked highly for MG-QOL15r in one NMA. The more recent network meta-analyses by Waseem et al. and McLaren et al. [44,45] further expanded the comparative evidence network by incorporating a broader range of targeted therapies and trials. Nevertheless, treatment rankings varied according to the outcome, treatment network, and analytical approach and should be interpreted cautiously because they were derived primarily from indirect comparisons across trials with different patient populations, baseline disease severity, concomitant therapies, outcome definitions, and follow-up periods. Therefore, probabilistic rankings should not be interpreted as evidence of definitive clinical superiority between individual agents or therapeutic classes.

The methodological concerns surrounding indirect treatment comparisons were also highlighted by Gilhus et al., [46] whose systematic literature review was used to evaluate the feasibility of indirect treatment comparisons in gMG. Their assessment identified cross-trial differences in study design, patient characteristics, background therapies, outcome assessment, and network connectivity that may affect the validity of comparative estimates. Although this review did not generate a new pooled comparative efficacy estimate, it provides complementary evidence regarding the interpretability of the treatment comparisons synthesized in the quantitative reviews.

Antibody status represents an additional source of clinical heterogeneity across the included evidence. Most of the evidence supporting complement inhibition was derived from predominantly AChR-antibody-positive populations, whereas some reviews also included MuSK-antibody-positive and seronegative patients. Serostatus was not consistently incorporated as a stratification factor in comparative analyses, despite differences in therapeutic response across antibody-defined subgroups. This is particularly relevant for the interpretation of rituximab, whose evidence base includes a greater representation of MuSK-antibody-positive patients in some reviews. Consequently, indirect comparisons across therapeutic mechanisms may partly reflect differences in the underlying serological populations rather than treatment effects alone.

The strength of comparative conclusions involving rituximab remains limited because direct head-to-head evidence against newer targeted therapies is scarce. Rituximab was included in this umbrella review as part of the broader therapeutic evidence landscape represented in the eligible systematic reviews, rather than as a mandatory direct comparator or to imply therapeutic equivalence across treatment mechanisms. Available comparisons are therefore predominantly indirect and involve trials differing in baseline disease severity, antibody status, prior and concomitant therapies, outcome definitions, and assessment timepoints. Evidence for rituximab in AChR-antibody-positive gMG remains heterogeneous, whereas more consistent benefit has been reported in MuSK-antibody-positive disease. Accordingly, the available secondary evidence does not support robust conclusions regarding superiority across therapeutic classes, and probabilistic rankings should be interpreted in the context of these clinical and methodological differences.

Methodological appraisal identified important limitations in the secondary evidence base. AMSTAR 2 rated only one review as high confidence, while four were rated as low confidence and five as critically low confidence. The most frequent critical weakness was the absence of a list of excluded studies with reasons (Domain 7: 8/10 reviews rated “No”). Protocol registration and prespecification were also inconsistent, with three reviews rated “No” and two rated “Partial Yes” for Domain 2. Reporting of primary-study funding sources was particularly limited (Domain 10: 9/10 reviews rated “No”), although this is not considered a critical AMSTAR 2 domain. These findings indicate that the apparent consistency of findings across reviews should be interpreted in the context of variable methodological rigor and limited confidence in a substantial proportion of the available secondary evidence.

Primary-study overlap was another major finding of this umbrella review. The overall corrected covered area (CCA) was 32.59%, indicating very high overlap, and 36 of the 45 pairwise review comparisons were also classified as having very high overlap (≥15%). No structural zeros were identified; therefore, adjustment for structural zeros did not alter the CCA. These findings demonstrate substantial reuse of the same primary trials across systematic reviews. Consequently, the apparent growth and consistency of the secondary evidence base does not necessarily represent independent replication of findings, but frequently reflects repeated synthesis of a relatively limited set of underlying trials. This distinction is particularly relevant in a rapidly evolving therapeutic field, where multiple systematic reviews and network meta-analyses may create an impression of greater evidentiary volume than actually exists. CCA was used solely to characterize overlap and inform interpretation, rather than as an eligibility criterion, because its magnitude may also be influenced by differences in the number of primary studies included across reviews [47].

The value of an umbrella review in this context differs from that of an updated systematic review of primary trials. Rather than generating another pooled estimate of treatment efficacy, the present study evaluates the methodological quality, independence, and consistency of the existing secondary evidence used to compare therapeutic options in gMG. By combining AMSTAR 2 appraisal with quantitative assessment of primary-study overlap, this review demonstrates that multiple published evidence syntheses should not automatically be interpreted as multiple independent confirmations of comparative efficacy.

From a clinical and implementation perspective, targeted therapies have demonstrated clinically meaningful improvements over placebo within relatively short trial periods, supporting their role in selected patients with gMG, particularly when rapid symptom control or reduction in treatment burden is clinically relevant. However, direct comparative evidence across therapeutic options remains limited, and cost, availability, treatment burden, and access continue to influence treatment decisions. These considerations reinforce the importance of specialist evaluation and shared decision-making, integrating disease severity, antibody status, prior treatment response, safety profile, route and frequency of administration, access, and patient preferences.

This umbrella review has several limitations. First, 10 systematic reviews and evidence syntheses met the predefined eligibility criteria, reflecting the intentionally focused search strategy requiring the therapeutic evidence framework to include rituximab. Although this approach was consistent with the prespecified review question, it may have excluded otherwise relevant systematic reviews of complement inhibitors or FcRn-targeted therapies that did not include rituximab within their therapeutic framework. Second, heterogeneity in antibody status, baseline disease severity, eligibility criteria, background therapies, outcome definitions, assessment timepoints, and follow-up periods limited cross-review comparability. In addition, the evidence syntheses included differed in their analytical purpose: most provided quantitative estimates of treatment effects, whereas the systematic review by Gilhus et al. [46] primarily informed an indirect treatment comparison feasibility assessment and therefore contributed evidence on the interpretability, rather than the magnitude, of comparative treatment effects. Third, our synthesis depended primarily on information reported by the included reviews, and antibody-specific subgroup data were not consistently available. Finally, the substantial primary-study overlap identified in our analysis should be interpreted as a characteristic of the current secondary evidence base rather than solely as a limitation of this umbrella review, as multiple reviews repeatedly synthesized many of the same underlying trials.

## 5. Conclusions

This umbrella review summarizes and critically appraises the current secondary evidence on complement inhibitors and FcRn-targeted therapies in generalized myasthenia gravis. Overall, the included evidence suggests that several targeted therapies are associated with improvements in disease-specific functional outcomes, particularly MG-ADL and QMG, although the magnitude and consistency of benefit vary across agents, populations, outcomes, and analytical approaches. Methodological confidence across the included reviews was heterogeneous, and the very high primary-study overlap indicates that the apparent volume of secondary evidence is not fully independent. In addition, differences in antibody-defined populations, cross-trial heterogeneity, and the predominantly indirect nature of comparative analyses limit robust conclusions regarding relative efficacy across individual agents or therapeutic classes. Therefore, probabilistic treatment rankings should not be interpreted as evidence of definitive clinical superiority. Future studies should prioritize antibody-stratified head-to-head comparisons, standardized outcome definitions and assessment timepoints, longer follow-up, robust safety evaluation, and economic analyses to better inform treatment selection, sequencing, and implementation, particularly in resource-limited healthcare systems such as those in Latin America.

## Figures and Tables

**Table 1:** Overlap assessment across included systematic reviews using the corrected covered area (CCA) method. CCA values were interpreted as slight (<5%), moderate (5% to <10%), high (10% to <15%), and very high (≥15%) overlap.

Overall Results		
Number of columns (number of reviews)	c	10
Number of rows (number of index publications)	r	30
Number of included primary studies (including double counting)	N	118
Covered area	N/(rc)	39.33%
Corrected covered area	(N − r)/(rc − r)	32.59%
Interpretation of overlap	Very High overlap	
Structural Zeros	X	0
Corrected covered area (adjusting by structural zeros)	(N − r)/(rc − r − X)	32.59%

## Data Availability

No new primary data were generated in this study. The data extracted and analyzed for this umbrella review, including methodological quality and primary-study overlap assessments, are available within the article and its Supplementary Materials.

## Supplementary Materials

The following supporting information can be downloaded at https://www.mdpi.com/article/10.3390/neurosci7050107/s1: Supplementary Text S1: Search strategy; Supplementary Text S2: Total references and duplicates; Supplementary Text S3: Grey literature; Supplementary Table S1: Studies excluded during the original search; Supplementary Table S2: Description of the included systematic reviews/PRISMA; Supplementary Table S3: Quality assessment of the included reviews according to AMSTAR 2; Supplementary Table S4: Corrected Coverage Area calculation (GROOVE).

## Abbreviations

AChR	Acetylcholine receptor
AChR-Ab+	Acetylcholine receptor antibody-positive
AMSTAR 2	A Measurement Tool to Assess Systematic Reviews 2
C5	Complement component 5
CCA	Corrected covered area
FcRn	Neonatal Fc receptor
FDA	U.S. Food and Drug Administration
gMG	Generalized myasthenia gravis
GROOVE	Graphical Representation of Overlap for Overviews
IgG	Immunoglobulin G
ITC	Indirect treatment comparison
JBI	Joanna Briggs Institute
LRP4	Low-density lipoprotein receptor-related protein 4
MeSH	Medical Subject Headings
MG	Myasthenia gravis
MG-ADL	Myasthenia Gravis Activities of Daily Living
MGC	Myasthenia Gravis Composite
MGFA	Myasthenia Gravis Foundation of America
MGFA-PIS	Myasthenia Gravis Foundation of America Post-Intervention Status
MG-QOL15r	15-item revised Myasthenia Gravis Quality of Life scale
MuSK	Muscle-specific kinase
NMA	Network meta-analysis
OSF	Open Science Framework
PICO	Population, Intervention, Comparator, and Outcome
PRISMA	Preferred Reporting Items for Systematic Reviews and Meta-Analyses
QMG	Quantitative Myasthenia Gravis
RCT	Randomized controlled trial
RTX	Rituximab
SR	Systematic review
