# Supplementary material for: Targeted Therapies in Generalized Myasthenia Gravis: An Umbrella Review of Evidence Quality and Overlap
Source: NeuroSci. 2026 Sep 30;7(5):107. doi: 10.3390/neurosci7050107 (PMC13708136; doi:10.3390/neurosci7050107)
Supplement: Supplementary file 1 [file neurosci-07-00107-s001.zip › Supplementary Material.pdf]

Online Supplementary Material

Targeted Therapies in Generalized Myasthenia Gravis: An Umbrella Review of Evidence Quality and Overlap

Castillo et al.

Supplementary File for the Umbrella Review. Contents:

Supplementary Text S1: Search strategy

Supplementary Text S2: Total references and duplicates

Supplementary Text S3: Grey Literature

Supplementary Table S1: Excluded list

Supplementary Table S2: Description of the included SRs/PRISMA

Supplementary Table S3: Quality assessment of the included reviews according to AMSTAR-2

Supplementary Table S4: Corrected Coverage Area calculation (GROOVE)

Supplementary Text S1: Search strategy

The original searches were conducted in April 2025. Prior to manuscript resubmission, the database searches were updated in August 2026 using the same search concepts and eligibility criteria. The updated searches covered the period from the day following the original search through the date of the update.

- ***PubMed***

Original search date: 25 April 2025

((("Myasthenia Gravis"[Mesh] OR "Myasthenia Gravis, Autoimmune, Experimental"[Mesh] OR Generalized Myasthenia Gravis OR Myastheni*) AND ("Complement Inactivating Agents"[Mesh] OR "Complement Inactivating Agents" [Pharmacological Action] OR "Antibodies, Monoclonal, Humanized"[Mesh] OR "eculizumab" [Supplementary Concept] OR Soliris OR 5G1.1 OR H5G1-1 OR H5G11 OR H5G1.1 OR H5G1.1VHC+H5G1.1VLC OR Alexion OR Elizaria OR "ravulizumab" [Supplementary Concept] OR ALXN1210 OR ALXN-1210 OR Ultomiris OR ALXN-1810 OR ALXN1810 OR "zilucoplan" [Supplementary Concept] OR zilbrysq OR "efgartigimod alfa" [Supplementary Concept] OR ARGX-113 OR Vyvgart OR "rozanolixizumab" [Supplementary Concept] OR UCB-7665 OR UCB7665 OR rystiggo OR nipocalimab OR batoclimab OR FcRn blockers OR FcRn inhibitors OR FcRn antagonist)) AND ("Rituximab"[Mesh] OR CD20 Antibody, Rituximab OR Rituximab CD20 Antibody OR Rituxan OR IDEC-C2B8 Antibody OR IDEC C2B8 Antibody OR IDEC-C2B8 OR IDEC C2B8 OR Mabthera OR GP2013 OR "Antibodies, Monoclonal"[Mesh])) AND (Systematic Review [Publication Type] OR Meta-Analysis [Publication Type])

History and Search Details — Download — Delete

Search	Actions	Details	Query	Results	Time
#5	•••	>	Search: ((("Myasthenia Gravis"[Mesh] OR "Myasthenia Gravis, Autoimmune, Experimental"[Mesh] OR Generalized Myasthenia Gravis OR Myastheni*) AND ("Complement Inactivating Agents"[Mesh] OR "Complement Inactivating Agents" [Pharmacological Action] OR "Antibodies, Monoclonal, Humanized"[Mesh] OR "eculizumab" [Supplementary Concept] OR Soliris OR 5G1.1 OR H5G1-1 OR H5G11 OR H5G1.1 OR H5G1.1VHC+H5G1.1VLC OR Alexion OR Elizaria OR "ravulizumab" [Supplementary Concept] OR ALXN1210 OR ALXN-1210 OR Ultomiris OR ALXN-1810 OR ALXN1810 OR "zilucoplan" [Supplementary Concept] OR zilbrysq OR "efgartigimod alfa" [Supplementary Concept] OR ARGX-113 OR Vyvgart OR "rozanolixizumab" [Supplementary Concept] OR UCB-7665 OR UCB7665 OR rystiggo OR nipocalimab OR batoclimab OR FcRn blockers OR FcRn inhibitors OR FcRn antagonist)) AND ("Rituximab"[Mesh] OR CD20 Antibody, Rituximab OR Rituximab CD20 Antibody OR Rituxan OR IDEC-C2B8 Antibody OR IDEC C2B8 Antibody OR IDEC-C2B8 OR IDEC C2B8 OR Mabthera OR GP2013 OR "Antibodies, Monoclonal"[Mesh])) AND (Systematic Review [Publication Type] OR Meta-Analysis [Publication Type])	15	13:42:41
#4	•••	>	Search: Systematic Review [Publication Type] OR Meta-Analysis [Publication Type]	376,375	13:38:51
#3	•••	>	Search: "Rituximab"[Mesh] OR CD20 Antibody, Rituximab OR Rituximab CD20 Antibody OR Rituxan OR IDEC-C2B8 Antibody OR IDEC C2B8 Antibody OR IDEC-C2B8 OR IDEC C2B8 OR Mabthera OR GP2013 OR "Antibodies, Monoclonal"[Mesh]	307,432	13:31:37
#2	•••	>	Search: "Complement Inactivating Agents"[Mesh] OR "Complement Inactivating Agents" [Pharmacological Action] OR "Antibodies, Monoclonal, Humanized"[Mesh] OR "eculizumab" [Supplementary Concept] OR Soliris OR 5G1.1 OR H5G1-1 OR H5G11 OR H5G1.1 OR H5G1.1VHC+H5G1.1VLC OR Alexion OR Elizaria OR "ravulizumab" [Supplementary Concept] OR ALXN1210 OR ALXN-1210 OR Ultomiris OR ALXN-1810 OR ALXN1810 OR "zilucoplan" [Supplementary Concept] OR zilbrysq OR "efgartigimod alfa" [Supplementary Concept] OR ARGX-113 OR Vyvgart OR "rozanolixizumab" [Supplementary Concept] OR UCB-7665 OR UCB7665 OR rystiggo OR nipocalimab OR batoclimab OR FcRn blockers OR FcRn inhibitors OR FcRn antagonist	120,537	13:30:43
#1	•••	>	Search: "Myasthenia Gravis"[Mesh] OR "Myasthenia Gravis, Autoimmune, Experimental"[Mesh] OR Generalized Myasthenia Gravis OR Myastheni*	24,461	13:29:36

Update period: 26 April 2025 to 15 August 2026

Search	Actions	Details	Query	Results	Time
#2	•••	>	Search: ((("Myasthenia Gravis"[Mesh] OR "Myasthenia Gravis, Autoimmune, Experimental"[Mesh] OR Generalized Myasthenia Gravis OR Myastheni*) AND ("Complement Inactivating Agents"[Mesh] OR "Complement Inactivating Agents" [Pharmacological Action] OR "Antibodies, Monoclonal, Humanized"[Mesh] OR "eculizumab" [Supplementary Concept] OR Soliris OR 5G1.1 OR H5G1-1 OR H5G11 OR H5G1.1 OR H5G1.1VHC+H5G1.1VLC OR Alexion OR Elizaria OR "ravulizumab" [Supplementary Concept] OR ALXN1210 OR ALXN-1210 OR Ultomiris OR ALXN-1810 OR ALXN1810 OR "zilucoplan" [Supplementary Concept] OR zilbrysq OR "efgartigimod alfa" [Supplementary Concept] OR ARGX-113 OR Vyvgart OR "rozanolixizumab" [Supplementary Concept] OR UCB-7665 OR UCB7665 OR rystiggo OR nipocalimab OR batoclimab OR FcRn blockers OR FcRn inhibitors OR FcRn antagonist)) AND ("Rituximab"[Mesh] OR CD20 Antibody, Rituximab OR Rituximab CD20 Antibody OR Rituxan OR IDEC-C2B8 Antibody OR IDEC C2B8 Antibody OR IDEC-C2B8 OR IDEC C2B8 OR Mabthera OR GP2013 OR "Antibodies, Monoclonal"[Mesh])) AND (Systematic Review [Publication Type] OR Meta-Analysis [Publication Type]) Filters: from 2025/4/26 - 2026/8/15 Sort by: Most Recent	8	19:50:28
#1	•••	>	Search: ((("Myasthenia Gravis"[Mesh] OR "Myasthenia Gravis, Autoimmune, Experimental"[Mesh] OR Generalized Myasthenia Gravis OR Myastheni*) AND ("Complement Inactivating Agents"[Mesh] OR "Complement Inactivating Agents" [Pharmacological Action] OR "Antibodies, Monoclonal, Humanized"[Mesh] OR "eculizumab" [Supplementary Concept] OR Soliris OR 5G1.1 OR H5G1-1 OR H5G11 OR H5G1.1 OR H5G1.1VHC+H5G1.1VLC OR Alexion OR Elizaria OR "ravulizumab" [Supplementary Concept] OR ALXN1210 OR ALXN-1210 OR Ultomiris OR ALXN-1810 OR ALXN1810 OR "zilucoplan" [Supplementary Concept] OR zilbrysq OR "efgartigimod alfa" [Supplementary Concept] OR ARGX-113 OR Vyvgart OR "rozanolixizumab" [Supplementary Concept] OR UCB-7665 OR UCB7665 OR rystiggo OR nipocalimab OR batoclimab OR FcRn blockers OR FcRn inhibitors OR FcRn antagonist)) AND ("Rituximab"[Mesh] OR CD20 Antibody, Rituximab OR Rituximab CD20 Antibody OR Rituxan OR IDEC-C2B8 Antibody OR IDEC C2B8 Antibody OR IDEC-C2B8 OR IDEC C2B8 OR Mabthera OR GP2013 OR "Antibodies, Monoclonal"[Mesh])) AND (Systematic Review [Publication Type] OR Meta-Analysis [Publication Type]) Sort by: Most Recent	23	19:49:43

- ***Embase***

Original search date: 25 April 2025

('myasthenia gravis'/syn OR 'generalized myasthenia gravis'/syn) AND ('complement inhibitor'/syn OR 'eculizumab'/syn OR 'ravulizumab'/syn OR 'zilucoplan'/syn OR 'fcrn inhibitor'/syn OR 'efgartigimod alfa'/syn OR 'rozanolixizumab'/syn OR 'nipocalimab'/syn OR 'batoclimab'/syn) AND ('rituximab'/syn) AND ([2015-2025]/py) AND ('systematic review'/syn OR 'meta analysis'/syn)

History Save | Delete | Print view | Export | Email Combine > using And Or Collapse

#7	#5 AND #6	21
#6	'systematic review'/syn OR 'meta analysis'/syn	835,156
#5	#1 AND #2 AND #3 AND [2015-2025]/py	291
#4	#1 AND #2 AND #3	321
#3	'rituximab'/syn	132,226
#2	'complement inhibitor'/syn OR 'eculizumab'/syn OR 'ravulizumab'/syn OR 'zilucoplan'/syn OR 'fcrn inhibitor'/syn OR 'efgartigimod alfa'/syn OR 'rozanolixizumab'/syn OR 'nipocalimab'/syn OR 'batoclimab'/syn	15,808
#1	'myasthenia gravis'/syn OR 'generalized myasthenia gravis'/syn	33,775

Update period: 26 April 2025 to 15 August 2026

('myasthenia gravis'/syn OR 'generalized myasthenia gravis'/syn) AND ('complement inhibitor'/syn OR 'eculizumab'/syn OR 'ravulizumab'/syn OR 'zilucoplan'/syn OR 'fcrn inhibitor'/syn OR 'efgartigimod alfa'/syn OR 'rozanolixizumab'/syn OR 'nipocalimab'/syn OR 'batoclimab'/syn) AND

'rituximab'/syn AND ('systematic review'/syn OR 'meta analysis'/syn) AND [26-04-2025]/sd NOT [16-08-2026]/sd

Enter search query e.g. 'cancer gene therapy'/exp OR ((treatment OR therapy) NEAR/5 fluorouracil):ab

('myasthenia gravis'/syn OR 'generalized myasthenia gravis'/syn) AND ('complement inhibitor'/syn OR 'eculizumab'/syn OR 'ravulizumab'/syn OR 'zilucoplan'/syn OR 'fcrn inhibitor'/syn OR 'efgartigimod alfa'/syn OR 'rozanolixizumab'/syn OR 'nipocalimab'/syn OR 'batoclimab'/syn) AND 'rituximab'/syn AND ('systematic review'/syn OR 'meta analysis'/syn) AND [26-04-2025]/sd NOT [16-08-2026]/sd

Mapping Date Fields Quick limits

Search history

Select all Combine using AND Save Delete Export Email

#1 ('myasthenia gravis'/syn OR 'generalized myasthenia gravis'/syn) AND ('complement inhibitor'/syn OR 'eculizumab'/syn OR 'ravulizumab'/syn OR 'zilucoplan'/syn OR 'fcrn inhibitor'/syn OR 'efgartigimod alfa'/syn OR 'rozanolixizumab'/syn OR 'nipocalimab'/syn OR 'batoclimab'/syn) AND 'rituximab'/syn 11

Show full

- ***Epistemonikos***

Original search date: 25 April 2025

(title:((title:((title:(myasthenia gravis) OR abstract:(myasthenia gravis)) OR (title:(myastheni*) OR abstract:(myastheni*)) OR (title:(generalized myasthenia gravis) OR abstract:(generalized myasthenia gravis))) OR abstract:((title:(myasthenia gravis) OR abstract:(myasthenia gravis)) OR (title:(myastheni*) OR abstract:(myastheni*)) OR (title:(generalized myasthenia gravis) OR abstract:(generalized myasthenia gravis))))) OR abstract:((title:((title:(myasthenia gravis) OR abstract:(myasthenia gravis)) OR (title:(myastheni*) OR abstract:(myastheni*)) OR (title:(generalized myasthenia gravis) OR abstract:(generalized myasthenia gravis))) OR abstract:((title:(myasthenia gravis) OR abstract:(myasthenia gravis)) OR (title:(myastheni*) OR abstract:(myastheni*)) OR (title:(generalized myasthenia gravis) OR abstract:(generalized myasthenia gravis)))))) AND (title:((title:(complement inhibitor) OR abstract:(complement inhibitor)) OR (title:(eculizumab) OR abstract:(eculizumab)) OR (title:(soliris) OR abstract:(soliris)) OR (title:(ravulizumab) OR abstract:(ravulizumab)) OR (title:(ultomiris) OR abstract:(ultomiris)) OR (title:(zilucoplan) OR abstract:(zilucoplan)) OR (title:(zilbrysq) OR abstract:(zilbrysq)) OR (title:(FcRn antagonist) OR abstract:(FcRn antagonist)) OR (title:(FcRn blockers) OR abstract:(FcRn blockers)) OR (title:(efgartigimod) OR abstract:(efgartigimod)) OR (title:(efgartigimod alfa) OR abstract:(efgartigimod alfa)) OR (title:(vyvgart) OR abstract:(vyvgart)) OR (title:(rozanolixizumab) OR abstract:(rozanolixizumab)) OR (title:(nipocalimab) OR abstract:(nipocalimab)) OR (title:(batoclimab) OR abstract:(batoclimab))) OR abstract:((title:(complement inhibitor) OR abstract:(complement inhibitor)) OR (title:(eculizumab) OR abstract:(eculizumab)) OR (title:(soliris) OR abstract:(soliris)) OR (title:(ravulizumab) OR abstract:(ravulizumab)) OR (title:(ultomiris) OR abstract:(ultomiris)) OR (title:(zilucoplan) OR abstract:(zilucoplan)) OR (title:(zilbrysq) OR abstract:(zilbrysq)) OR (title:(FcRn antagonist) OR abstract:(FcRn antagonist)) OR (title:(FcRn blockers) OR abstract:(FcRn blockers)) OR (title:(efgartigimod) OR abstract:(efgartigimod)) OR (title:(efgartigimod alfa) OR abstract:(efgartigimod alfa)) OR (title:(vyvgart) OR abstract:(vyvgart)) OR (title:(rozanolixizumab) OR abstract:(rozanolixizumab)) OR (title:(nipocalimab) OR abstract:(nipocalimab)) OR (title:(batoclimab) OR abstract:(batoclimab)))) AND

(title:((title:(rituximab) OR abstract:(rituximab)) OR (title:(rituxan) OR abstract:(rituxan)) OR (title:(mabthera) OR abstract:(mabthera))) OR abstract:((title:(rituximab) OR abstract:(rituximab)) OR (title:(rituxan) OR abstract:(rituxan)) OR (title:(mabthera) OR abstract:(mabthera))))

Filter: Last 10 years

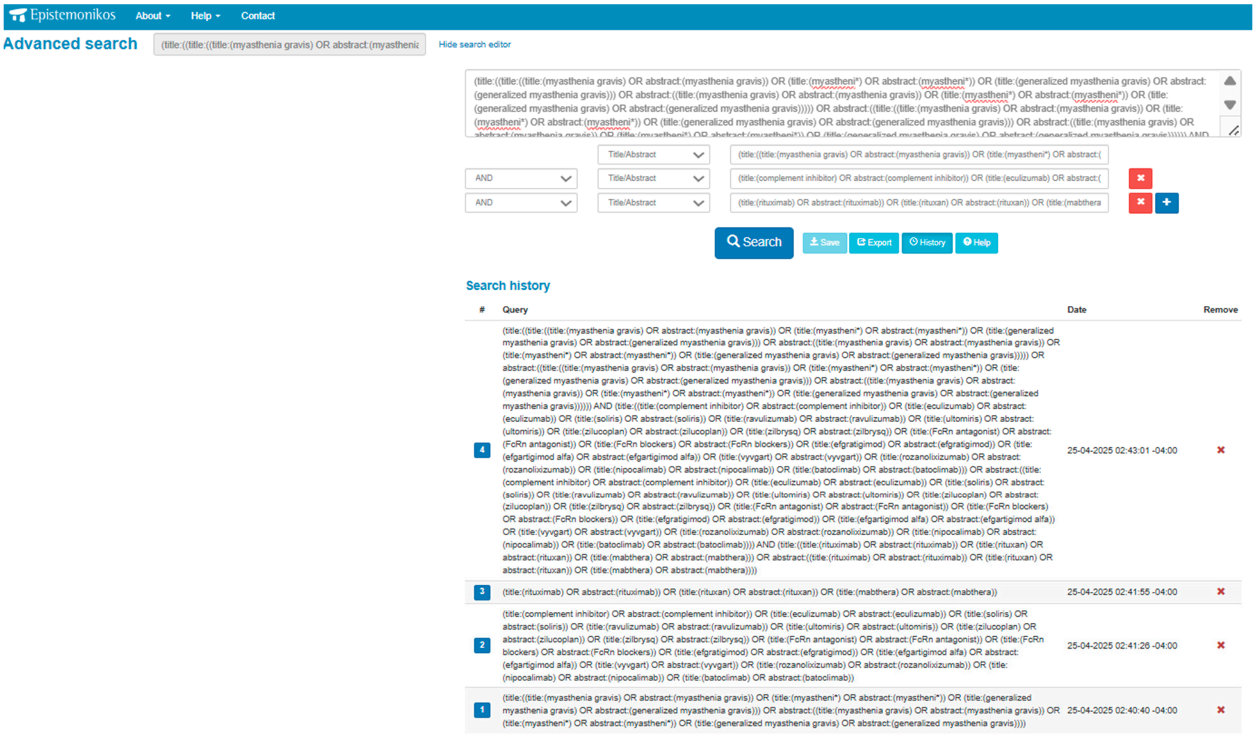

Update period: 2025 to 2026

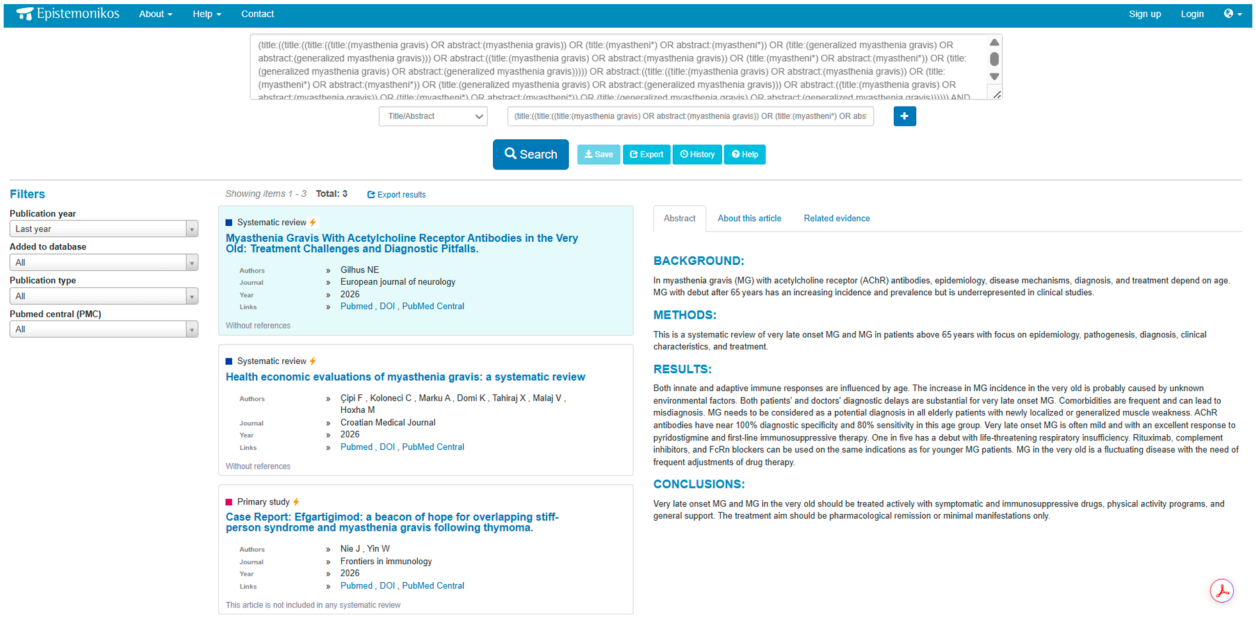

Supplementary Text S2: Total references and duplicates

Original search

A total of 51 references were imported from databases: 15 from PubMed, 21 from Embase, and 15 from Epistemonikos. After removing 11 duplicates within database records, 40 unique database records remained. Seven additional records were identified through supplementary searches, of which one overlapped with database records. Therefore, 46 unique records were screened.

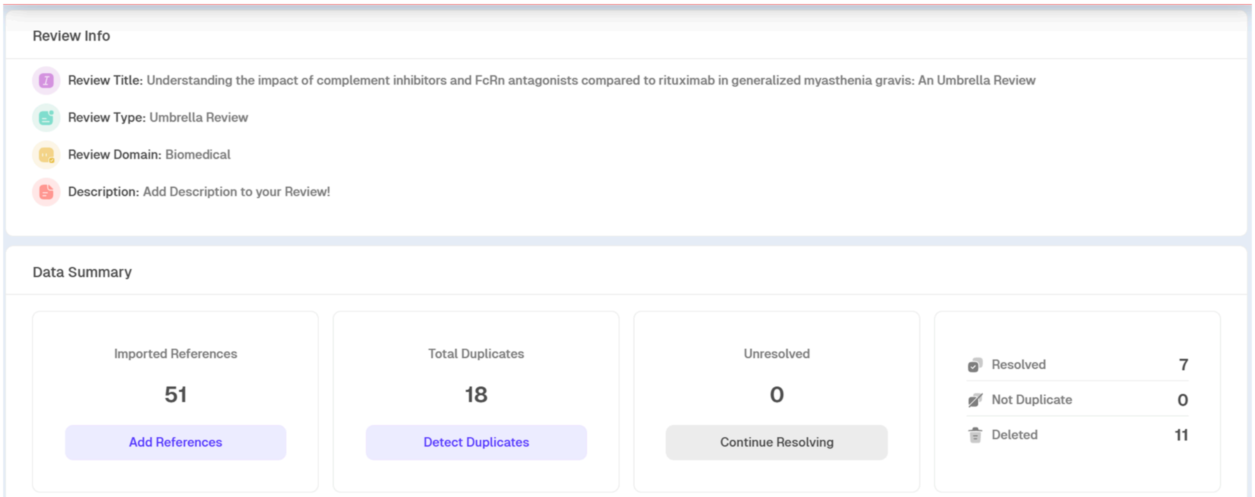

Updated search

Prior to manuscript resubmission, an updated database search was conducted in August 2026 using the same predefined search strategy and eligibility criteria. A total of 22 additional records were retrieved from the three electronic databases: 8 from PubMed, 11 from Embase, and 3 from Epistemonikos. After removing 7 duplicate records, 15 unique database records remained. Of these, 2 records had already been identified in the original search and were therefore removed from the updated screening set. The remaining 13 new records underwent title and abstract screening; 11 were excluded at this stage and 2 reports were selected for full-text assessment (1,2).

Supplementary Text S3: Grey Literature

- ***Google Scholar***

myasthenia gravis AND (eculizumab OR ravulizumab OR zilucoplan OR efgartigimod OR nipocalimab OR rozanolixizumab) AND rituximab AND (systematic review OR meta-analysis)

Original search

Of the 5 pages reviewed, 3 articles are of interest (3–5).

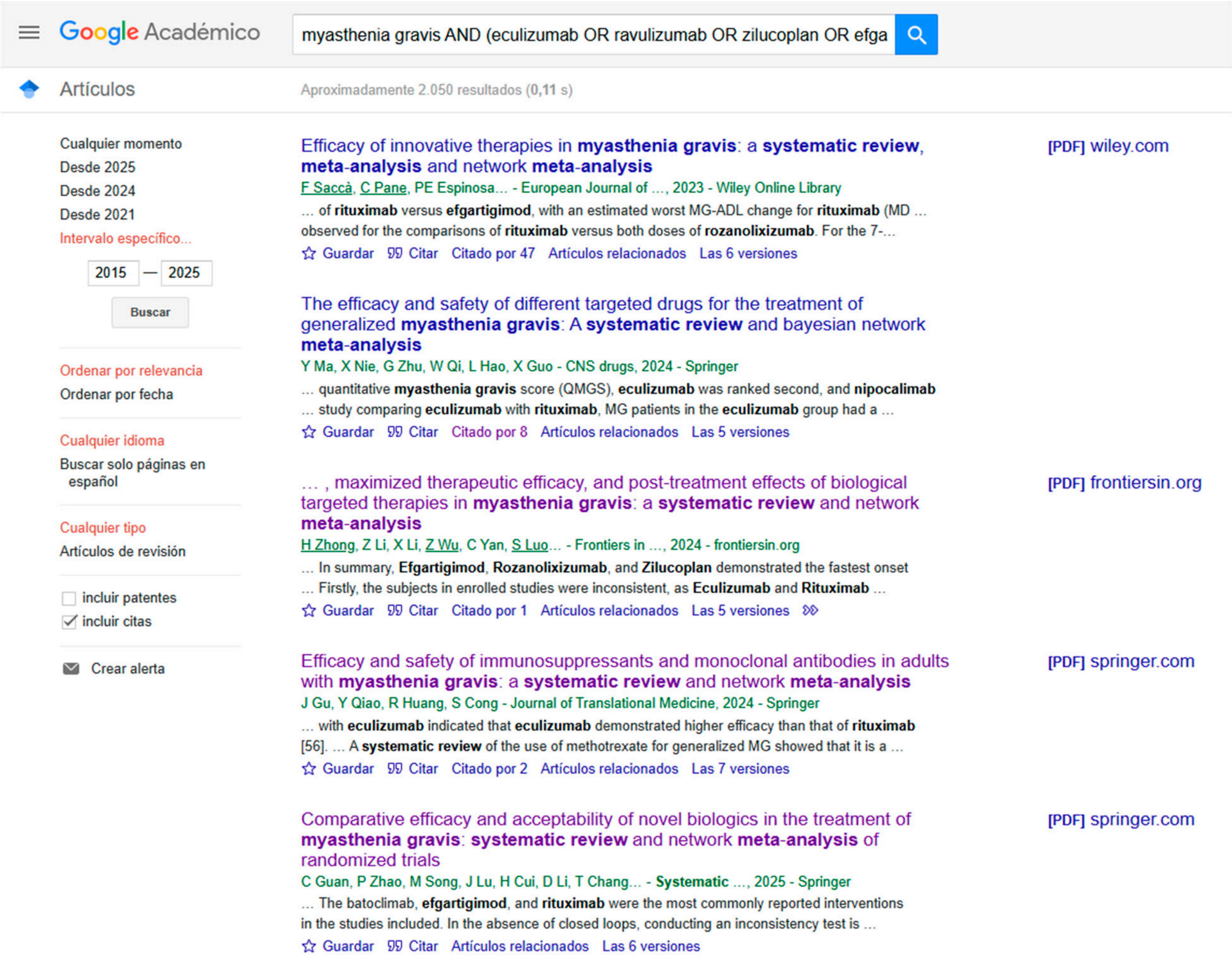

In addition, one potentially eligible report was identified through the updated Google Scholar search and was also assessed at full text using the same predefined eligibility criteria (6).

- ***Research Rabbit***

After conducting database searches and selecting articles, 7 peer-reviewed articles were uploaded to the Research Rabbit platform, where an exploration of similar works was conducted. Following the search, 686 related articles were obtained, which were subsequently filtered using the concepts of 'systematic review', resulting in 4 papers (7–10). Research Rabbit was not repeated as part of the 2026 search update.

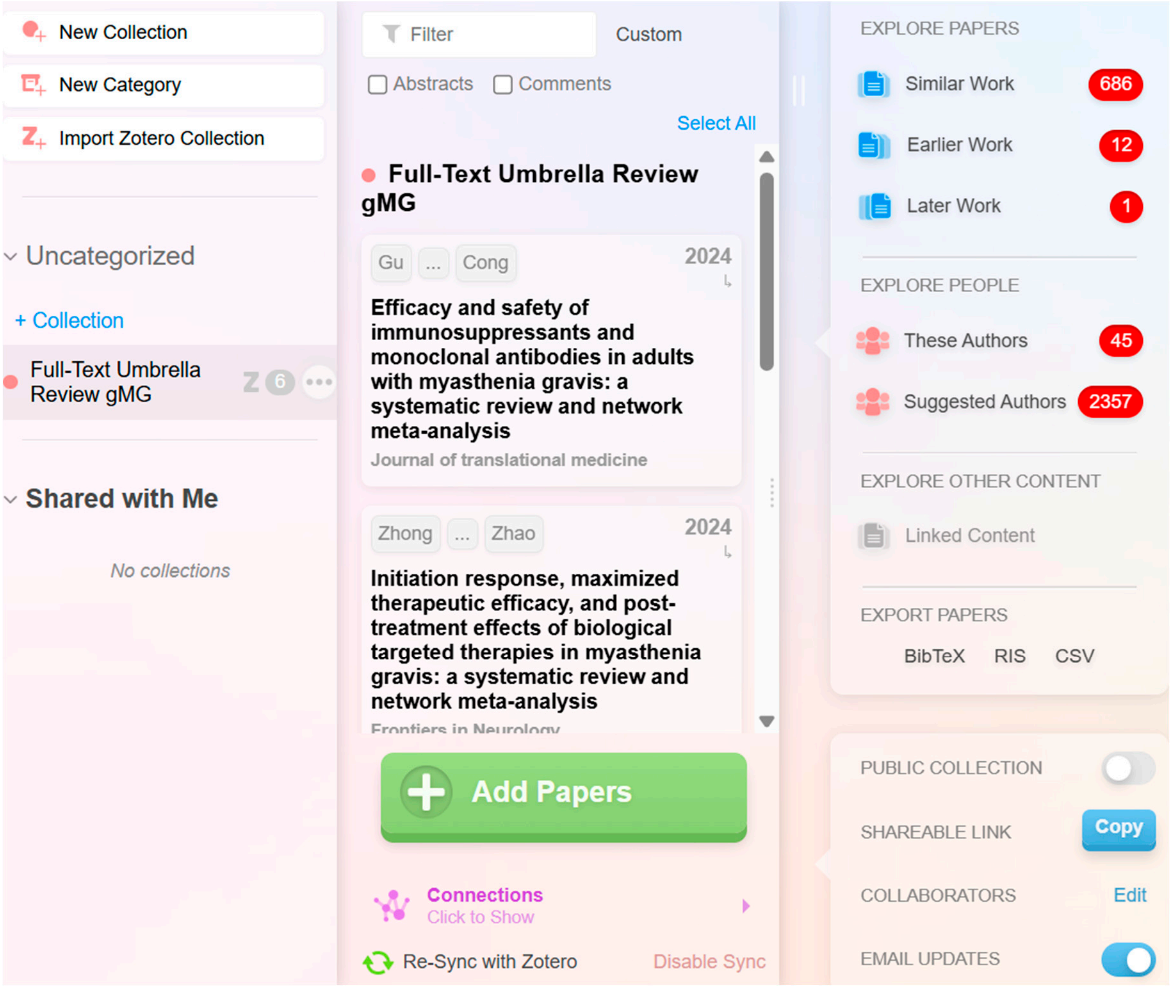
New Collection
New Category
Import Zotero Collection
Uncategorized
+ Collection
Full-Text Umbrella Review gMG
Shared with Me
No collections
Filter
Custom
Abstracts
Comments
Select All
Full-Text Umbrella Review gMG
Gu ... Cong 2024
Efficacy and safety of immunosuppressants and monoclonal antibodies in adults with myasthenia gravis: a systematic review and network meta-analysis
Journal of translational medicine
Zhong ... Zhao 2024
Initiation response, maximized therapeutic efficacy, and post-treatment effects of biological targeted therapies in myasthenia gravis: a systematic review and network meta-analysis
Frontiers in Neurology
Add Papers
Connections
Click to Show
Re-Sync with Zotero
Disable Sync
EXPLORE PAPERS
Similar Work 686
Earlier Work 12
Later Work 1
EXPLORE PEOPLE
These Authors 45
Suggested Authors 2357
EXPLORE OTHER CONTENT
Linked Content
EXPORT PAPERS
BibTeX
RIS
CSV
PUBLIC COLLECTION
SHAREABLE LINK
Copy
COLLABORATORS
Edit
EMAIL UPDATES

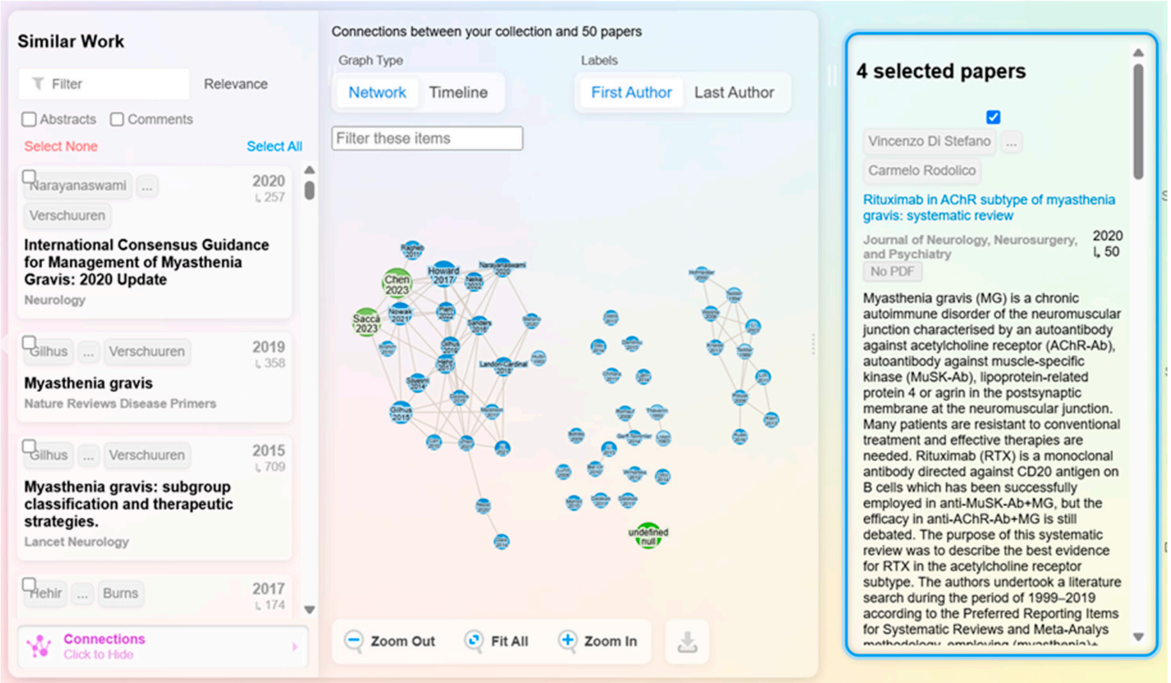
Similar Work
Filter
Relevance
Abstracts
Comments
Select None
Select All
Narayanaswami ... Verschuuren 2020
International Consensus Guidance for Management of Myasthenia Gravis: 2020 Update
Neurology
Gilhus ... Verschuuren 2019
Myasthenia gravis
Nature Reviews Disease Primers
Gilhus ... Verschuuren 2015
Myasthenia gravis: subgroup classification and therapeutic strategies.
Lancet Neurology
Hehir ... Burns 2017
Connections
Click to Hide
Connections between your collection and 50 papers
Graph Type
Network
Timeline
Labels
First Author
Last Author
Filter these items
Zoom Out
Fit All
Zoom In
4 selected papers
Vincenzo Di Stefano ...
Carmelo Rodolico
Rituximab in AChR subtype of myasthenia gravis: systematic review
Journal of Neurology, Neurosurgery, and Psychiatry
2020
No PDF
Myasthenia gravis (MG) is a chronic autoimmune disorder of the neuromuscular junction characterised by an autoantibody against acetylcholine receptor (AChR-Ab), autoantibody against muscle-specific kinase (MuSK-Ab), lipoprotein-related protein 4 or agrin in the postsynaptic membrane at the neuromuscular junction. Many patients are resistant to conventional treatment and effective therapies are needed. Rituximab (RTX) is a monoclonal antibody directed against CD20 antigen on B cells which has been successfully employed in anti-MuSK-Ab+MG, but the efficacy in anti-AChR-Ab+MG is still debated. The purpose of this systematic review was to describe the best evidence for RTX in the acetylcholine receptor subtype. The authors undertook a literature search during the period of 1999–2019 according to the Preferred Reporting Items for Systematic Reviews and Meta-Analys

Supplementary Table S1: Studies excluded during the original search

Year	Author	Title	DOI	Exclude reason
2024	Smith, A. Gordon et al.	Risk-Benefit Analysis of Novel Treatments for Patients with Generalized Myasthenia Gravis.	10.1007/s12325-024-03014-5	Not included RTX
2024	Li, Jiaxuan et al.	The efficacy and safety of FcRn inhibitors in patients with myasthenia gravis: a systematic review and meta-analysis.	10.1007/s00415-024-12247-x	Not included RTX
2021	Xiao, Hai et al.	Clinical Efficacy and Safety of Eculizumab for Treating Myasthenia Gravis.	10.3389/fimmu.2021.715036	Not a systematic review
2021	Kaegi, Celine et al.	Systematic review of safety and efficacy of belimumab in treating immune-mediated disorders.	10.1111/all.14704	Not included population
2019	Johansen, Annette et al.	Neuromuscular adverse events associated with anti-PD-1 monoclonal antibodies: Systematic review.	10.1212/WNL.0000000000007235	Not included population
2022	Bixio, Riccardo et al.	Rheumatoid arthritis and myasthenia gravis: a case-based review of the therapeutic options.	10.1007/s10067-022-06062-w	Not a systematic review
2019	Wang, Liang et al.	Immunosuppressive and monoclonal antibody treatment for myasthenia gravis: A network meta-analysis.	10.1111/cns.13110	Not included RTX
2024	Otto, Ferdinand et al.	Beyond T cell toxicity - Intrathecal chemokine CXCL13 indicating B cell involvement in immune-related adverse events following checkpoint inhibition: A two-case series and literature review.	10.1111/ene.16279	Not a systematic review
2022	Barišić, N. et al.	Vaccination in pediatric acquired inflammatory immune-mediated neuromuscular disorders	10.1016/j.ejpn.2021.12.014	Not a systematic review
2022	Gelinas, D. et al.	The humanistic burden of myasthenia gravis: A systematic literature review	10.1016/j.jns.2022.120268	Not a systematic review
2022	Gomathy, S.B. et al.	Molecular Therapy in Myasthenia Gravis	10.17925/USN.2022.18.1.49	Not a systematic review
2023	Hiew, F.L.	ACHR.Positive Generalized Myasthenia Gravis: The Old is Gold and the New Is for us to Explore	10.4103/aian.aian_430_23	Not a systematic review
2020	Huda, R.	New Approaches to Targeting B Cells for Myasthenia Gravis Therapy	10.3389/fimmu.2020.00240	Not a systematic review
2016	Melzer, N. et al.	Clinical features, pathogenesis, and treatment of myasthenia gravis: a supplement to the Guidelines of the German Neurological Society	10.1007/s00415-016-8045-z	Not a systematic review
2023	Morrison, A.H. et al.	Ocular Myasthenia Gravis	10.1007/s11940-023-00753-8	Not a systematic review
2022	Nabizadeh, F. et al.	Autologous hematopoietic stem cell transplantation in neuromyelitis optica spectrum disorder: A systematic review and meta-analysis	10.1016/j.jocn.2022.08.020	Not included population
2020	Pastor, S. et al.	Efficacy and safety of non-steroidal immunosuppressive treatments in Generalized Myasthenia Gravis patients. A systematic review and meta-analysis	N/A	Abstract for a conference paper, no data
2021	Phillips, G.A. et al.	PND27 Cost-of-Illness for Adults with Generalized Myasthenia Gravis in the US	10.1016/j.jval.2021.04.811	Not a systematic review
2023	Santana, E. et al.	Ablative Treatment of Inflammatory and Other Diseases with Monoclonal Antibodies (Mabs) or Janus Kinases (Nibs) Increases Risk for Infection	10.1097/IPC.0000000000001317	Not a systematic review
2022	Su, Y. et al.	Knowledge mapping of targeted immunotherapy for myasthenia gravis from 1998 to 2022: A bibliometric analysis	10.3389/fimmu.2022.998217	Not a systematic review

2023	Tozza, S. et al.	Value of Antibody Determinations in Chronic Dysimmune Neuropathies	10.3390/brainsci13010037	Not a systematic review
2023	Vesperinas-Castro, A. et al.	Rituximab treatment in myasthenia gravis	10.3389/fneur.2023.1275533	Not a systematic review
2023	Xu, Z. et al.	Development of Therapeutic Proteins for a New Subcutaneous Route of Administration After the Establishment of Intravenous Dosages: A Systematic Review	10.1002/cpt.2823	Not a systematic review
2024	Zulfiqar, F. et al.	Outcomes with chimeric antigen receptor T-cell therapy in Rheumatological disorders: A systematic review	10.1016/j.trim.2024.102137	Not a systematic review
2021	Siddiqi ZA et al.	Eculizumab in refractory generalized myasthenia gravis previously treated with rituximab: subgroup analysis of REGAIN and its extension study.	10.1002/mus.27422	Not a systematic review
2019	Evoli, A.	Myasthenia Gravis: An update	10.1080/14737175.2019.1600404	Not a systematic review
2022	Nelke C et al.	Eculizumab versus rituximab in generalised myasthenia gravis.	10.1136/jnnp-2021-328665	Not a systematic review
2020	Gagnon, A.	Suboptimal Control of Generalized Myasthenia revealed by implementation of systematic follow-up in a French- Canadian Community neurology practice	10.1212/WNL.0000000000010209	Not a systematic review
2017	Howard JF et al.	Safety and efficacy of eculizumab in anti-acetylcholine receptor antibody-positive refractory generalised myasthenia gravis (REGAIN): a phase 3, randomised, double-blind, placebo-controlled, multicentre study.	10.1016/S1474-4422(17)30369-1	Not a systematic review
2023	Wartmann H et al.	Incidence, prevalence, hospitalization rates and treatment patterns in myasthenia gravis: A 10-year real-world data analysis of German claims data.	10.1159/000529583	Not a systematic review
2024	Okusa S et al.	Serious Bacterial Infections Associated with Eculizumab: A Pharmacovigilance Study.	10.2169/internalmedicine.1893-23	Not a systematic review
2024	Puerta C et al.	Autoimmune Encephalitis Induced by SARS-CoV-2 Infection Treated with Thymectomy.	10.1016/j.atssr.2024.04.035	Not a systematic review
2021	Patti, F. et al.	Efficacy and safety of eculizumab in patients with treatment-refractory anti-acetylcholine receptor antibody-positive generalised myasthenia gravis previously treated with rituximab	10.1016/j.jns.2021.118339	Not a systematic review
2024	Alhamood, Mohammed et al.	Efficacy of Recent Immunotherapies in Myasthenia Gravis: A Systematic Review (2019-2024)	10.21203/rs.3.rs-4851265/v1	Preprints Have Not Been Peer Reviewed
2023	Suryadevara, Vineet et al.	Comparing Safety and Efficacy of Rituximab and Eculizumab in Treating Refractory Myasthenia Gravis: A Systematic Review	10.56570/jimgs.v2i2.138	Narrative Review
2021	Zhao, Cong et al.	Effectiveness and Safety of Rituximab for Refractory Myasthenia Gravis: A Systematic Review and Single-Arm Meta-Analysis	10.3389/fneur.2021.736190	Not included Complement Inhibitors OR FcRn Blockers
2021	Li, Ting et al.	Efficacy and safety of different dosages of rituximab for refractory generalized AChR myasthenia gravis: A meta-analysis	10.1016/j.jocn.2020.11.043	Not included Complement Inhibitors OR FcRn Blockers
2020	Di Stefano, Vincenzo et al.	Rituximab in AChR subtype of myasthenia gravis: systematic review	10.1136/jnnp-2019-322606	Not included Complement Inhibitors OR FcRn Blockers
2017	Tandan, Rup et al.	Rituximab treatment of myasthenia gravis: A systematic review	10.1002/mus.25597	Not included Complement Inhibitors OR FcRn Blockers

Supplementary Table S2: is provided as a separate Excel file.

Supplementary Table S3: Quality assessment of the included reviews according to AMSTAR-2

Author	Year	DOI	1	2	3	4	5	6	7	8	9	10	11	12	13	14	15	16	AMSTAR-2
Gu	2024	10.1186/s12967-024-05751-1	Y	Y	Y	Y	Y	Y	N	Y	Y	N	Y	Y	Y	Y	Y	Y	Low
Ma	2024	10.1007/s40263-024-01062-7	Y	N	Y	Y	Y	Y	N	Y	Y	Y	Y	Y	Y	Y	Y	Y	Critically Low
Zhong	2024	10.3389/fneur.2024.1479685	Y	N	Y	P	Y	Y	N	Y	Y	N	Y	Y	Y	Y	Y	Y	Critically Low
Saccà	2023	10.1111/ene.15872	Y	N	Y	Y	Y	Y	N	Y	Y	N	Y	Y	Y	Y	N	Y	Critically Low
Chen	2023	10.3389/fimmu.2023.1280226	Y	Y	Y	Y	Y	Y	Y	Y	Y	N	Y	Y	Y	Y	Y	Y	High
Feng	2021	10.3389/fneur.2021.725700	Y	Y	Y	P	Y	Y	Y	Y	P	N	Y	N	N	Y	Y	Y	Low
Guan	2025	10.1186/s13643-025-02859-3	Y	Y	Y	P	Y	Y	N	Y	Y	N	Y	Y	Y	Y	Y	Y	Low
Gilhus	2025	10.57264/cer-2025-0009	Y	P	Y	P	Y	Y	N	Y	P	N	X	X	Y	Y	X	Y	Low
Waseem	2026	10.1002/brb3.71557	Y	P	N	P	Y	Y	N	P	Y	N	Y	N	N	Y	N	Y	Critically Low
McLaren	2026	10.1212/WNL.0000000000218351	Y	Y	N	P	Y	Y	N	P	Y	N	Y	N	N	Y	N	Y	Critically Low

Critical Domain

N= no, does not fulfill the criteria
P = partial yes, fulfills primary criteria
Y = yes, fulfills criteria
X = not applicable

1 PICO
2 Protocol
3 Explanation for study designs included
4 Search strategy
5 Duplicate study selection
6 Duplicate data extraction
7 List of excluded studies
8 Study data details
9 Risk of bias assessment
10 Primary study funders
11 Metanalysis method
12 Primary study bias impact on meta-analysis
13 Primary study bias impact on findings
14 Heterogeneity
15 Publication bias
16 Authors' conflict of interests

Supplementary Table S4: Corrected Coverage Area calculation (GROOVE). (11)

Primary Studies		Systematic Reviews (Mandatory)									
**Study ID (Mandatory)**	**Reference (Optional)**	**Zhong H 2024**	**Ma Y 2024**	**Gu 2024**	**Feng X 2021**	**Chen 2023**	**Saccà 2023**	**Guan 2025**	**Gilhus 2025**	**Waseem 2026**	**McLaren 2026**
Howard et al. 2013	ECULIZUMAB		1		1						1
Howard et al. 2017	ECULIZUMAB (REGAIN)	1	1	1	1	1	1	1	1	1	1
Howard et al. 2019	EFGARTIGIMOD		1	1		1		1	1	1	1
Bril et al. 2021	ROZANOLIXIZUMAB		1	1				1	1	1	1
Howard et at. 2021	EFGARTIGIMOD (ADAPT)	1	1	1		1	1	1	1	1	1
Oyama et al. 2020	ECULIZUMAB				1						
Levine et al. 2019	ECULIZUMAB				1						
Mantegazza et al. 2021	ECULIZUMAB (MGFA-PIS)				1						
Govindarajan et al. 2020	ECULIZUMAB				1						
Anderson 2016	RTX (MMT)				1						
Choi 2019	RTX (MGFA-PIS)				1						
Jhing 2019	RTX				1						
Topakian 2019	RTX (MGFA-PIS)				1						
Landon-Cardinal 2018	RTX				1						
Robeson 2017	RTX (MGFA-PIS)				1						
Litchman 2020	RTX (MGFA-PIS)				1						
Brauner 2020	RTX				1						
Antozzi 2024	NIPOCALIMAB	1		1		1			1	1	1
Bril et al. 2022/2023	ROZANOLIXIZUMAB (MycarinG)	1		1		1	1		1	1	1
Yan et al. 2022	BATOCLIMAB	1	1	1		1		1		1	1
Nowak et al. 2024	BATOCLIMAB	1									1
Yan et al. 2024	BATOCLIMAB	1		1				1		1	1
Howard et al. 2020	ZILUCOPLAN	1	1	1				1	1	1	1
Howard el at. 2022/2023	ZILUCOPLAN (RAISE)	1		1		1	1		1	1	1
Vu et al. 2022	RAVULIZUMAB (CHAMPION)	1	1	1		1	1	1	1	1	1
Piehl et al. 2022	RTX (RINOMAX)	1	1	1		1	1	1	1	1	1
Nowak et al. 2021	RTX (BeatMG)		1	1		1	1	1	1	1	1
Guptill et al. 2021	NIPOCALIMAB		1								
Howard et al., 2024	EFGARTIGIMOD (ADAPT-SC)								1		
Antozzi 2025	NIPOCALIMAB									1	1

1. Waseem MH, Abideen ZU, Iqbal M, Ishtiaq S, Thada PK, Dmytriw AA. Comparing Efficacy and Safety of Various Monoclonal Antibodies in Myasthenia Gravis: A Systematic Review and Network Meta-Analysis of Randomized Controlled Trials. Brain Behav. 2026 Jun;16(6):e71557. doi:10.1002/brb3.71557 PubMed PMID: 42304705; PubMed Central PMCID: PMC13272886.

2. Gilhus NE, Jacob S, Hashim M, Van Sanden S, Drudge C, Nero A, et al. Network connectivity, between-study heterogeneity and timepoint challenges in generalized myasthenia gravis: a feasibility assessment of indirect treatment comparisons. J Comp Eff Res. 2025;14(6). Located at: Embase. doi:10.57264/cer-2025-0009

3. Chang Guan, Peixi Zhao, Meijin Song, Jing Lu, Huijing Cui, Dongxu Li, et al. Comparative efficacy and acceptability of novel biologics in the treatment of myasthenia gravis: systematic review and network meta-analysis of randomized trials. Syst Rev. 2025. doi:10.1186/s13643-025-02859-3 PubMed PMID: 40346603; PubMed Central PMCID: 12063394.

4. Suryadevara V, Habab Y, Hutcheson A, Panjiyar B, Davydov G, Nashat H, et al. Comparing Safety and Efficacy of Rituximab and Eculizumab in Treating Refractory Myasthenia Gravis: A Systematic Review. J Int Med Grad. 2023;2(2).

5. Alhamood M, Suleiman HAD, Saadeh I, Abbass A. Efficacy of Recent Immunotherapies in Myasthenia Gravis: A Systematic Review (2019-2024). 2024.

6. McLaren NP, Rosati M, Zhong W, Hussain S, Funaro MC, Nowak RJ, et al. Comparison of the Efficacy and Safety of Myasthenia Gravis Treatments. Neurology. 2026 Aug 11;107(3):e218351. doi:10.1212/WNL.0000000000218351

7. Zhao C, Pu M, Chen D, Shi J, Li Z, Guo J, et al. Effectiveness and Safety of Rituximab for Refractory Myasthenia Gravis: A Systematic Review and Single-Arm Meta-Analysis. Front Neurol. 2021;Volume 12-2021. doi:10.3389/fneur.2021.736190

8. Li T, Zhang GQ, Li Y, Dong SA, Wang N, Yi M, et al. Efficacy and safety of different dosages of rituximab for refractory generalized AChR myasthenia gravis: A meta-analysis. J Clin Neurosci. 2021 Mar 1;85:6–12. doi:10.1016/j.jocn.2020.11.043

9. Di Stefano V, Lupica A, Rispoli MG, Di Muzio A, Brighina F, Rodolico C. Rituximab in AChR subtype of myasthenia gravis: systematic review. J Neurol Neurosurg Psychiatry. 2020;91(4):392–5. doi:10.1136/jnnp-2019-322606

10. Tandan R, Hehir II MK, Waheed W, Howard DB. Rituximab treatment of myasthenia gravis: A systematic review. Muscle Nerve. 2017;56(2):185–96. doi:https://doi.org/10.1002/mus.25597

11. Bracchiglione J, Meza N, Bangdiwala SI, Niño de Guzmán E, Urrútia G, Bonfill X, et al. Graphical Representation of Overlap for OVErviews: GROOVE tool. Res Synth Methods. 2022 May 1;13(3):381–8. doi:10.1002/jrsm.1557
